# Integrative multi-region MRI radiomics and clinical nomogram for preoperative lymphovascular invasion prediction in rectal cancer: a multicenter validation

**DOI:** 10.1186/s12880-025-02105-1

**Published:** 2025-12-19

**Authors:** Tianxian Chen, Ru Yi, Zhifeng Liu, Qing Chen, Wei Yuan, Qiangqiang Zhou

**Affiliations:** 1 Department of Medical Imaging, Ganzhou People’s Hospital, The Affiliated Ganzhou Hospital of Nanchang University, Ganzhou, 341000 China; 2 Department of Medical Imaging, The Fourth Affiliated Hospital of Guangzhou Medical University, Guangzhou, 511300 China; 3 Department of Pathology, Ganzhou People’s Hospital, The Affiliated Ganzhou Hospital of Nanchang University, Ganzhou, 341000 China

**Keywords:** Rectal cancer, Habitat, Radiomics, Nomogram

## Abstract

**Objective:**

This study aims to construct a nomogram using habitat radiomics, radiomics, and clinical features derived from multiparametric MRI (mpMRI) to improve the accuracy of preoperative prediction of lymphovascular invasion (LVI) status in rectal cancer patients.

**Methods:**

Data from 372 pathologically confirmed rectal cancer cases were retrospectively collected from two centers. Data from Center 1 were randomly split in a 7:3 ratio into a training cohort (*n* = 201) and an internal validation cohort (*n* = 87). Data from Center 2 served as external validation (*n* = 84). K-means clustering was used to divide the tumor into three subregions. Radiomics features were extracted from regions of interest and selected, and three machine learning algorithms were employed to construct radiomics models. A nomogram was created by integrating clinical, radiomics, and habitat radiomics features. The model’s predictive accuracy was assessed using AUC metrics, while practical clinical applicability was evaluated via calibration plots and decision curve analysis. SHAP values were employed to measure the contribution of individual radiomic features to predictive outcomes, thereby offering transparent insights for clinical decision-making processes.

**Results:**

The nomogram model outperformed other single models in predicting LVI, with AUCs of 0.978, 0.909, and 0.889 in the training, validation, and external test sets, respectively. Compared with the intratumoral model alone, the nomogram model achieved improvements of 20.3%, 14.1%, and 13.8%, respectively.

**Conclusion:**

The nomogram model developed in this study significantly improved the accuracy of predicting preoperative lymphovascular invasion status in rectal cancer.

**Supplementary Information:**

The online version contains supplementary material available at 10.1186/s12880-025-02105-1.

## Background

Colorectal cancer is the third most common malignancy worldwide, with a continuously rising incidence, particularly in younger populations under 50 years of age [1]. Lymphovascular invasion (LVI) denotes the pathological process whereby malignant tumor cells infiltrate into blood vessels and/or lymphatic channels. This phenomenon is a recognized indicator of aggressive tumor behavior and a crucial early step in metastatic progression in rectal cancer [2]. LVI is an important prognostic indicator in rectal cancer. In a study involving 330 patients, the detection rate of LVI was approximately 32.1% [3]. Extensive evidence indicates that colorectal cancer patients exhibiting LVI experience significantly poorer outcomes, with reductions in overall survival (OS) and disease-free survival (DFS) relative to those without LVI. Moreover, LVI correlates strongly with advanced tumor stage and low differentiation grade, and is routinely considered when determining postoperative adjuvant therapy and surveillance strategies [4–6]. Given its significant impact on prognosis, precise preoperative identification of LVI is essential for tailoring therapeutic regimens. Traditional assessment of LVI in rectal cancer depends on histopathological review of biopsy or surgical specimens, often resulting in detection only after resection and missing the window for neoadjuvant intervention. Therefore, there is a critical need for noninvasive, preoperative technologies capable of reliably determining LVI status to inform individualized treatment planning and enhance clinical outcomes.

Although high-resolution magnetic resonance imaging (MRI) remains the gold standard for preoperative staging and postoperative follow-up in rectal carcinoma, conventional MRI sequences lack sufficient sensitivity to reliably identify lymphovascular invasion [7, 8]. Radiomics captures a broad spectrum of quantitative image descriptors, reshaping raw scans into data-driven indicators that mirror the underlying complexity of tumor biology [9, 10]. Multiple studies have applied predictive models based on radiomics features to forecast preoperative lymphovascular invasion in rectal cancer, demonstrating promising predictive performance [7, 11, 12]. However, these studies primarily focus on intratumoral features, while the features of the peritumoral region, which may contain critical information, have not been fully explored [13–15]. Tumor heterogeneity extends beyond malignant cells to include stromal and infiltrating immune populations in the peritumoral milieu, which critically influence tumor proliferation, invasion, and metastatic dissemination [16–18].

Habitat analysis segments medical images into functionally relevant subregions by clustering quantitative imaging features, providing a data-driven approach to characterize heterogeneous tumor microenvironments. While the clustering process is based on imaging signal patterns (intensity and texture similarity), the resulting subregions have been shown to capture underlying biological heterogeneity that correlates with tissue composition, cellularity, necrosis, and vascular characteristics [19, 20]. By partitioning tumors into phenotypically distinct subregions, habitat analysis refines spatial heterogeneity assessment beyond whole-tumor radiomics. Importantly, habitat-based radiomic features have demonstrated predictive value for clinically relevant endpoints including neoadjuvant chemoradiotherapy response, metastasis, staging, and survival outcomes in locally advanced rectal cancer, suggesting that these imaging-derived subregions reflect biologically meaningful tumor characteristics [21–23]. However, its role in assessing LVI remains underexplored and warrants further investigation.

This study intends to construct a nomogram by integrating habitat features derived from multiparametric MRI, intratumoral and peritumoral radiomic features, and clinical variables to enhance the accuracy of preoperative prediction of lymphovascular invasion in rectal cancer.

## Materials and methods

### Study population

**Fig. 1:**
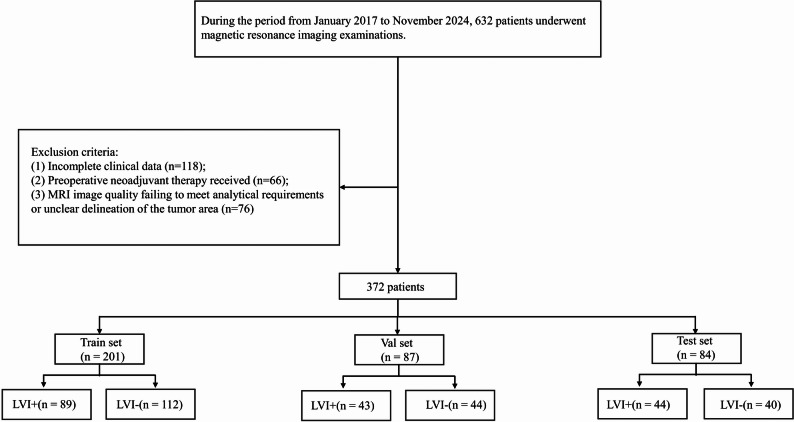
The flow chart shows the patient recruitment pathway

This retrospective study was approved by the primary institutional review board, which also granted a waiver of informed consent. Clinical and imaging data were collected from all patients with pathologically confirmed rectal cancer who underwent surgery at Centers 1 and 2 between January 2017 and November 2024. Inclusion criteria were: (1) postoperative pathological confirmation of rectal cancer; (2) completion of MRI within two weeks before surgery; (3) no history of other malignancies; and (4) availability of complete postoperative pathological reports and clinical data. Exclusion criteria were: (1) incomplete clinical data (*n* = 118); (2) receipt of neoadjuvant therapy before surgery (*n* = 66); and (3) MRI image quality inadequate for analysis or unclear tumor delineation (*n* = 76). A total of 372 rectal cancer (RC) patients were included in this study, divided into three cohorts: a training cohort (*n* = 201), an internal validation cohort (*n* = 87), and an external validation set (*n* = 84). The inclusion/exclusion criteria and screening process are illustrated in Fig. 1.

### Histopathological evaluation combined with lymphovascular invasion status analysis

Lymphovascular invasion is confirmed when postoperative histology reveals tumor cells within endothelial-lined channels or identifies intravascular tumor thrombi; absence of these features denotes LVI negativity.

### Magnetic resonance imaging protocol

Preoperative MRI was conducted on Siemens Skyra and GE 3.0 T platforms, with analysis focused on two axial protocols: high-resolution T2-weighted imaging and diffusion-weighted imaging. Detailed acquisition parameters are catalogued in the supplementary appendix.

### Image acquisition and segmentation

DICOM images were retrieved from the institutional archive and standardized in ITK-SNAP v3.6.0 using grayscale normalization. Volumes were resampled to 1 × 1 × 1 mm³ voxels to harmonize spatial resolution. On axial T2WI, two radiologists each with over ten years of oncologic imaging experience manually delineated tumor ROIs in ITK-SNAP. Diffusion-weighted images were then affine-registered to T2WI via SimpleITK’s registration module (https://simpleitk.org/). Peritumoral masks were generated automatically at 2 mm, 2–4 mm, and 4–6 mm offsets to systematically assess the impact of varying margin sizes on model performance. To evaluate intra-observer reproducibility, 30 cases were re-segmented by one radiologist after a two-week interval; features with intraclass correlation coefficients above 0.75 were deemed robust and retained. We extracted 19 radiomic features from each voxel within the tumor ROI to comprehensively capture local signal intensity and texture variations. K-means clustering was subsequently applied to these voxel-level features to establish a unified clustering scheme across all patients and ensure consistent label assignment. For detailed information regarding the specific features and clustering methodology, please refer to the Supplementary Materials. The optimal number of clusters was determined to be three based on the Silhouette score.

### Feature extraction and model construction

**Fig. 2:**
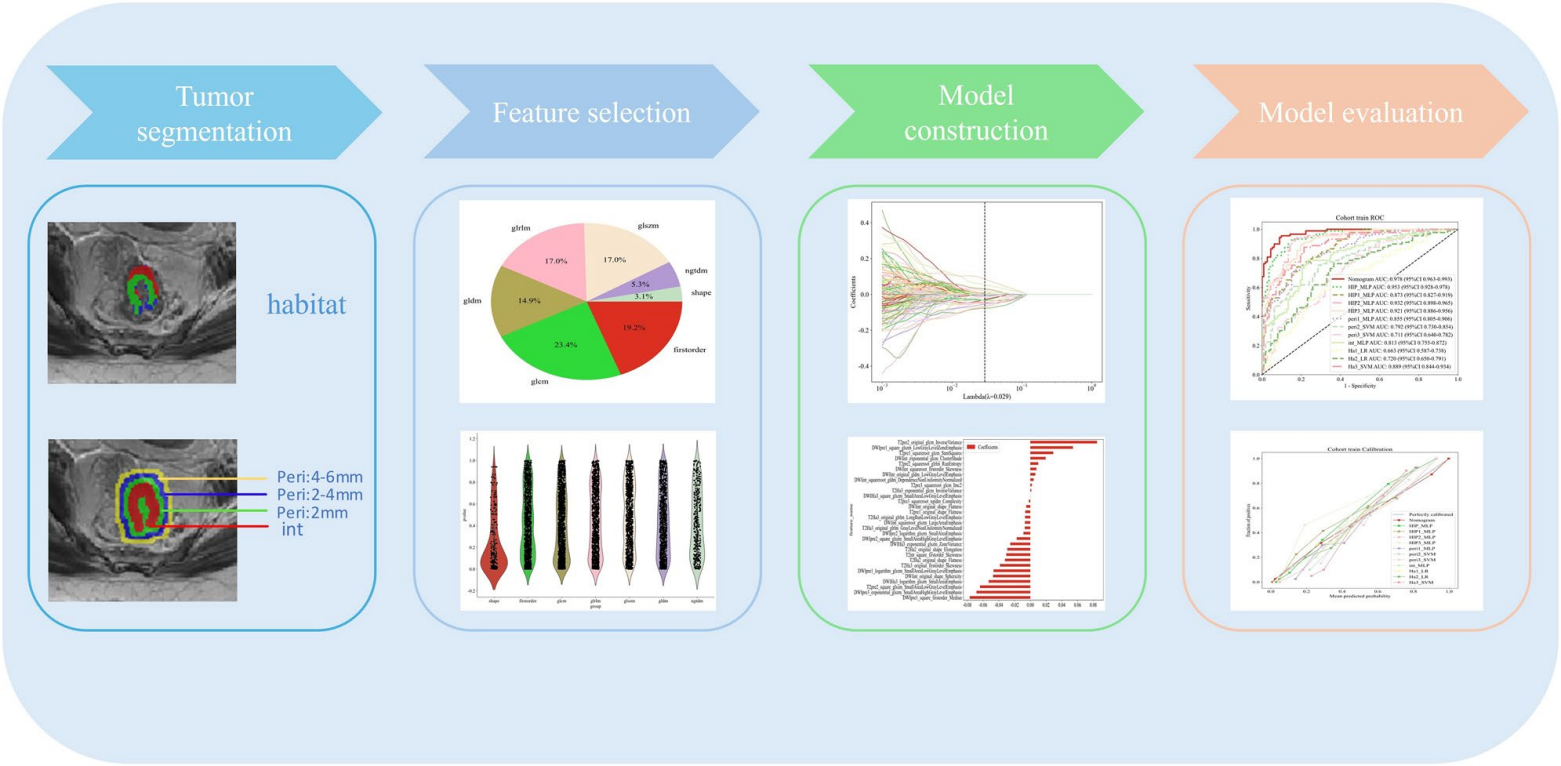
The overall workflow of this study

This study followed the Image Biomarker Standardization Initiative (IBSI) guidelines and extracted a total of 938 radiomics features from T2 and DWI sequences using the PyRadiomics library, including shape, first-order statistics, and texture features. Considering the potential systematic bias introduced by variations in scanners and imaging parameters across multiple centers, we applied the ComBat harmonization method to the extracted radiomic features, followed by Z-score normalization. The ComBat method, based on an empirical Bayes framework and originally developed for genomic data integration, effectively removes batch effects while preserving true biological information. This correction was performed prior to feature normalization to minimize scanner-related discrepancies, thereby enhancing the consistency of radiomic features across centers and improving the generalizability of the predictive model [24–26]. Significant features (*p* < 0.05) were selected by the Mann–Whitney U test. To prevent model overfitting, the minimum redundancy maximum relevance (mRMR) method was first applied to evaluate the correlation between features and the target variable, and low-correlation features were excluded. Subsequently, least absolute shrinkage and selection operator (LASSO) regression with 10-fold cross-validation was performed to determine the optimal λ value and identify the final feature set. The features selected through the mRMR and LASSO procedures were fed into the machine learning algorithms. Subsequently, the SHAP method was applied to these selected original features to interpret their contributions to the model’s predictions and enhance its explainability. The detailed workflow is shown in Fig. 2. The model construction process in this study systematically integrated multi-source information. Radiomic features were extracted from the intratumoral region (Int) and from peritumoral regions extending outward from the tumor boundary by 2 mm (Peri1), 2–4 mm (Peri2), and 4–6 mm (Peri3), and four corresponding predictive models were established based on these independent feature sets. In addition, three habitat radiomics models (Ha1, Ha2, and Ha3) were constructed using features derived from three distinct radiomics habitat subregions. For clinical variable analysis, univariate and multivariate logistic regression analyses were performed to identify independent clinical predictors (N stage) associated with lymphovascular invasion (LVI) status. Based on the selected radiomic features, three machine learning algorithms—logistic regression (LR), support vector machine (SVM), and multilayer perceptron (MLP)—were employed to develop predictive models, and their performances were systematically compared to determine the optimal approach. To further improve predictive accuracy, four multi-scale fusion models—HIP1, HIP2, HIP3, and HIP—were developed by integrating intratumoral radiomics and all habitat features with Peri1, Peri2, Peri3, and all peritumoral features, respectively, to systematically evaluate the impact of varying margin sizes on model performance. To determine the best hyperparameter settings for each algorithm and reduce overfitting, we implemented a grid search strategy with five-fold cross-validation within the training dataset. The internal validation cohort was used to select the best algorithm among LR, SVM, and MLP. Model robustness and predictive capability for lymphovascular invasion were then evaluated on the external test set. The detailed hyperparameter values optimized during training are provided in the Supplementary Materials (S2). Finally, to promote clinical translation and provide individualized risk assessment, a nomogram was developed by integrating the independent clinical predictor (N stage) with the best-performing HIP model in the internal validation set using a weighted linear combination, offering an intuitive and quantitative decision-support tool for preoperative LVI risk evaluation. The composition factors and corresponding coefficients of the nomogram are presented in the Supplementary Materials (S4).

### Model evaluation and statistical analysis

In this study, continuous variables are presented as mean ± standard deviation (SD), while categorical variables are reported as frequencies and percentages (n, %). Differences between groups were assessed using the independent samples t-test or the Mann-Whitney U test, depending on the data distribution. All statistical analyses were performed using Python software (version 3.7). Receiver operating characteristic (ROC) curves were used to evaluate the discriminative performance of the models, and the DeLong test was employed to statistically compare the areas under the curves (AUC) between different models. Decision curve analysis (DCA) was used to quantify and compare the clinical utility of each model. Additionally, calibration curves were plotted to assess the agreement between the predicted probabilities and the actual outcomes, thereby testing the models’ reliability. To enhance model interpretability, SHAP analysis was conducted to quantify the contribution of each feature to the HIP3 model’s predictions and to further elucidate the influence of the tumor microenvironment on the LVI status. In all statistical tests, a two-tailed P-value of less than 0.05 was considered statistically significant.

## Results

### Patients and clinical characteristics

A total of 372 patients were included in this study, divided into an LVI-positive group (*n* = 176) and an LVI-negative group (*n* = 196) based on their lymphovascular invasion (LVI) status. The baseline clinical characteristics of the patients, including age, sex, T stage, N stage, carcinoembryonic antigen (CEA), hematochezia, and hypertension, are summarized in Table 1. Statistical comparisons of all clinical characteristics among the training, internal validation, and external testing cohorts revealed no significant differences (all *P* > 0.05), indicating the comparability of baseline data across the cohorts. Univariate and multivariate logistic regression analyses were conducted on the complete clinical baseline data (Table 2). The analysis revealed that N stage remained statistically significant in both the univariate and multivariate analyses (*P* < 0.05) and were identified as independent risk factors for LVI.

**Table 1:** Baseline patient demographics and clinical characteristics

Characteristics	-label = ALL	-label = train	-label = val	-label = test	pvalue
Age	62.952 ± 11.106	64.189 ± 11.050	61.701 ± 11.424	61.286 ± 10.664	0.064
Sex					0.111
Female	150(40.323)	77(38.308)	31(35.632)	42(50.000)	
Male	222(59.677)	124(61.692)	56(64.368)	42(50.000)	
CEA					0.347
0–5 ng/mL	288(77.419)	154(76.617)	72(82.759)	62(73.810)	
≥ 5 ng/mL	84(22.581)	47(23.383)	15(17.241)	22(26.190)	
FOBT					0.458
Negative	195(52.419)	109(54.229)	47(54.023)	39(46.429)	
Positive	177(47.581)	92(45.771)	40(45.977)	45(53.571)	
HBP					0.270
Negative	286(76.882)	153(76.119)	72(82.759)	61(72.619)	
Positive	86(23.118)	48(23.881)	15(17.241)	23(27.381)	
T-stage					0.331
1	24(6.452)	12(5.970)	10(11.494)	2(2.381)	
2	102(27.419)	56(27.861)	23(26.437)	23(27.381)	
3	237(63.710)	127(63.184)	53(60.920)	57(67.857)	
4	9(2.419)	6(2.985)	1(1.149)	2(2.381)	
N-stage					0.359
0	221(59.409)	126(62.687)	44(50.575)	51(60.714)	
1	92(24.731)	44(21.891)	26(29.885)	22(26.190)	
2	59(15.860)	31(15.423)	17(19.540)	11(13.095)	

CEA carcinoembryonic antigen; FOBT fecal occult blood test; HBP high blood pressure

**Table 2:** Univariate and multivariate logistic regression analysis results

Characteristics	OR_UNI	OR lower 95%CI_UNI	OR upper 95%CI_UNI	p_value_UNI	OR_MULTI	OR lower 95%CI_MULTI	OR upper 95%CI_MULTI	p_value_MULTI
HBP	0.655	0.403	1.065	0.152				
FOBT	0.878	0.623	1.237	0.532				
Sex	0.879	0.654	1.182	0.473				
T-stage	0.967	0.887	1.053	0.515				
Age	0.997	0.993	1.000	0.125				
CEA	1.136	0.703	1.839	0.662				
N-stage	4.470	2.790	7.156	<0.001*	4.470	2.790	7.156	<0.001*

* *P* < 0.05

### Comparison of the performance of different models

**Fig. 3:**
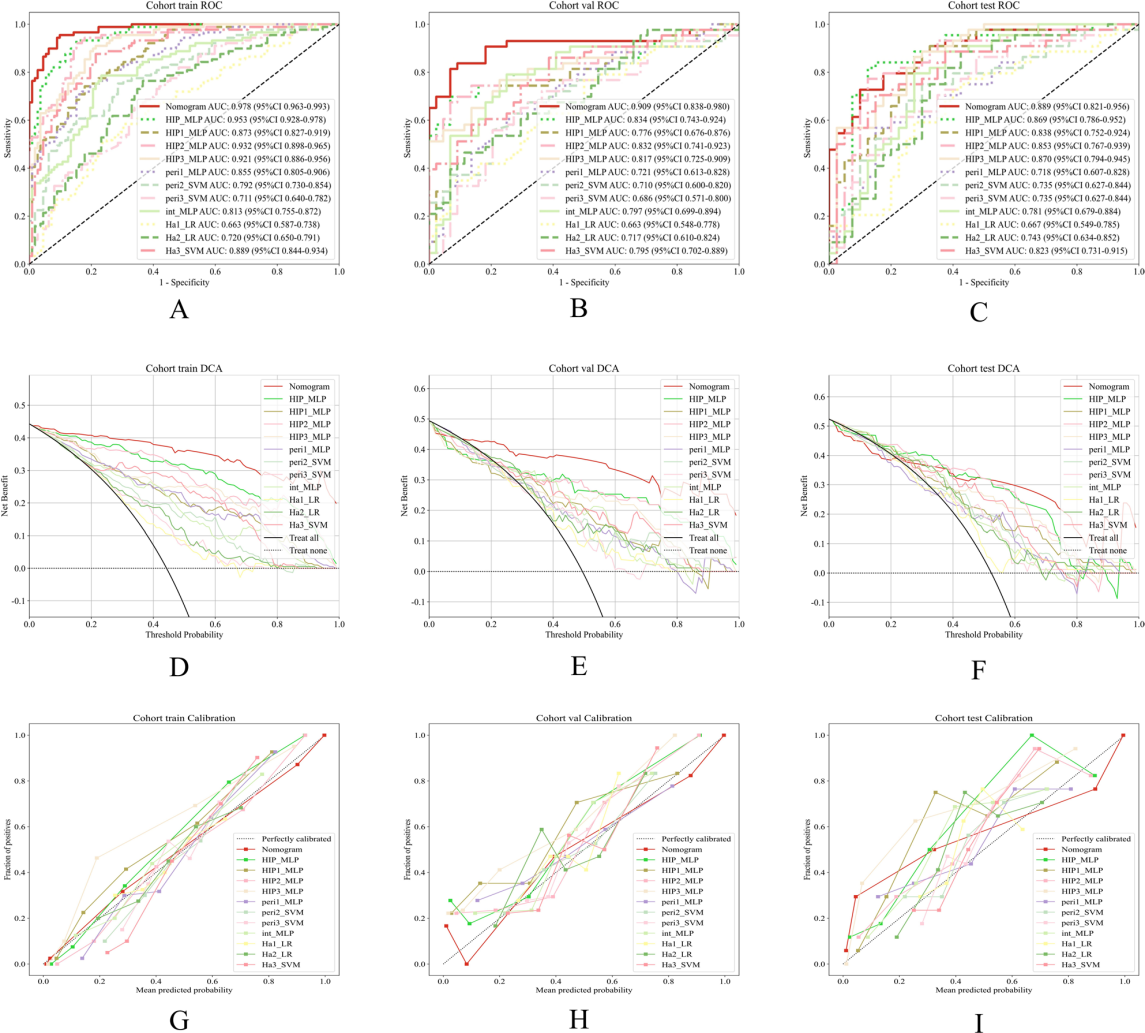
Comparative analysis of different models across the training, validation, and test cohorts: (**A**–**C**) ROC curve analysis (AUC); (**D**–**F**) decision curve analysis; (**G**–**I**) calibration curve analysis

**Fig. 4:**
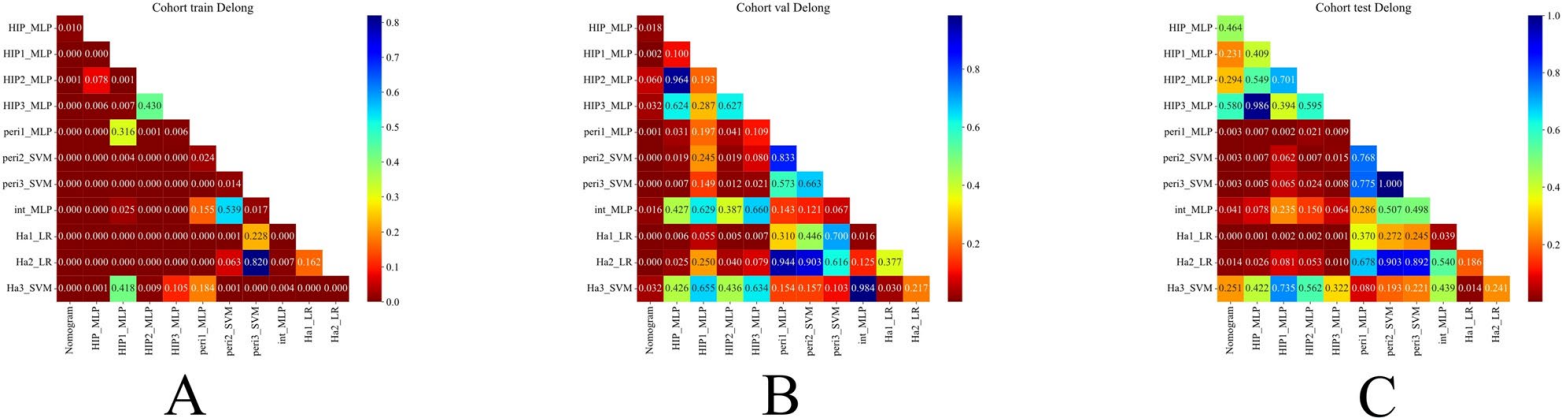
DeLong test results comparing the classification performance of different models for LVI across the training set (**A**), validation set (**B**), and test set (**C**)

**Fig. 5:**
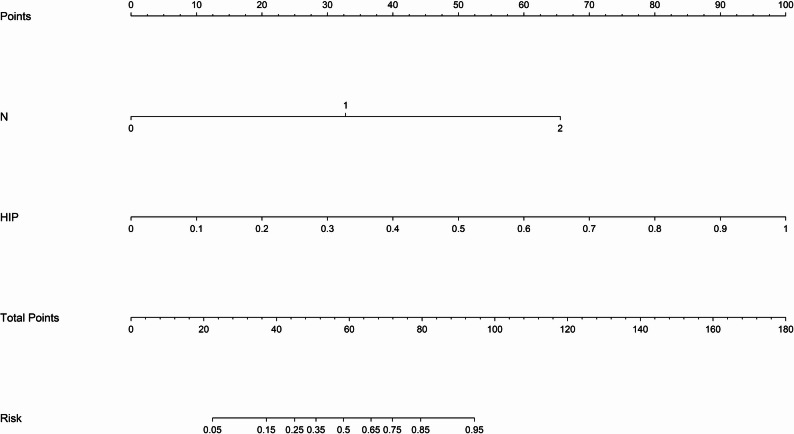
Nomogram developed for clinical application

Table 3; Fig. 3 (A–C) present the detailed performance metrics of the predictive model with the optimally performing algorithm in the internal validation set. Among the subregional radiomics models, the Ha3 model consistently demonstrated superior performance across different cohorts. In the training cohort, it achieved an AUC of 0.889 and an accuracy of 0.826, both higher than those of Ha1 (AUC 0.663, accuracy 0.647) and Ha2 (AUC 0.720, accuracy 0.692). In the internal validation cohort, Ha3 maintained favorable discriminative ability (AUC 0.795, accuracy 0.736), outperforming Ha1 (AUC 0.663, accuracy 0.644) and Ha2 (AUC 0.717, accuracy 0.678). In the external test cohort, Ha3 achieved an AUC of 0.823 and an accuracy of 0.774, again surpassing Ha1 (AUC 0.667, accuracy 0.667) and Ha2 (AUC 0.743, accuracy 0.738). These results suggest that Ha3 exhibits greater stability and generalizability in capturing tumor heterogeneity. For the radiomics models, the Int model achieved superior performance in the internal validation cohort (AUC 0.797, accuracy 0.770) and external test cohort (AUC 0.781, accuracy 0.762), compared with the peritumoral models (Pre1–3: AUC range 0.686–0.735, accuracy 0.678–0.726). Among all fusion models, the HIP2 model performed better than HIP1 and HIP3 models in the internal validation set (AUC 0.832), while the HIP1 model showed the poorest performance. HIP demonstrated the most outstanding predictive performance. In the training cohort, it achieved an AUC of 0.953 (95% CI: 0.9276–0.9785), 0.834 in the validation cohort (95% CI: 0.7435–0.9235), and 0.869 in the external test cohort (95% CI: 0.7863–0.9524). This consistent and robust performance across datasets was significantly superior to that of single-feature models, indicating that the HIP model, which integrates intratumoral, peritumoral, and habitat radiomic features, can substantially enhance the prediction of LVI in rectal cancer. Its stable performance in the external test cohort further underscores its potential for clinical application. The decision curve analyses (Fig. 3D–F) and calibration curves (Fig. 3G–I) demonstrated that the nomogram model achieved good calibration and provided net clinical benefit in the assessment of vascular invasion, indicating its significant clinical value. We assessed the statistical significance of AUC differences among the three HIP algorithms using the DeLong test, as shown in Supplementary Fig. 2. The results indicate comparable performance among the three algorithms on both the internal validation and external test sets (pairwise comparisons all *p* > 0.05), with MLP achieving the highest AUC in the internal validation set. Therefore, to optimize predictive performance and clinical applicability, we ultimately selected the MLP algorithm to construct the nomogram. The DeLong test (Fig. 4) showed that, among all models, the nomogram (Fig. 5) model had the highest diagnostic value. In the training cohort, the nomogram model performed significantly better than the other models, with statistically significant differences (*p* < 0.05). In both the internal validation cohort and the external test set, the nomogram model also demonstrated notable improvements compared with the Int, Peri1, Peri2, Peri3, Ha1, and Ha2 models (*p* < 0.05). Detailed performance metrics and comparative analysis results of the other models across the training cohort, internal validation cohort, and external test cohort are presented in the Supplementary Materials (S3).

**Table 3:** Detailed performance metrics of the predictive models

Signature	Accuracy	AUC	95% CI	Sensitivity	Specificity	PPV	NPV	Precision	Recall	F1	Threshold	Cohort
Nomogram	0.925	0.978	0.9632–0.9931	0.955	0.902	0.885	0.962	0.885	0.955	0.919	0.258	train
HIP_MLP	0.886	0.953	0.9276–0.9785	0.933	0.848	0.830	0.941	0.830	0.933	0.878	0.291	train
HIP1_MLP	0.786	0.873	0.8269–0.9191	0.742	0.821	0.767	0.800	0.767	0.742	0.754	0.353	train
HIP2_MLP	0.881	0.932	0.8983–0.9648	0.921	0.848	0.828	0.931	0.828	0.921	0.872	0.417	train
HIP3_MLP	0.841	0.921	0.8862–0.9557	0.910	0.786	0.771	0.917	0.771	0.910	0.835	0.168	train
peri1_MLP	0.786	0.855	0.8050–0.9059	0.708	0.848	0.787	0.785	0.787	0.708	0.746	0.491	train
peri2_SVM	0.731	0.792	0.7297–0.8539	0.753	0.714	0.677	0.784	0.677	0.753	0.713	0.453	train
peri3_SVM	0.652	0.711	0.6402–0.7818	0.820	0.518	0.575	0.784	0.575	0.820	0.676	0.396	train
int_MLP	0.776	0.813	0.7545–0.8723	0.787	0.768	0.729	0.819	0.729	0.787	0.757	0.396	train
Ha1_LR	0.647	0.663	0.5874–0.7384	0.584	0.696	0.605	0.678	0.605	0.584	0.594	0.465	train
Ha2_LR	0.692	0.720	0.6497–0.7911	0.764	0.634	0.624	0.772	0.624	0.764	0.687	0.425	train
Ha3_SVM	0.826	0.889	0.8441–0.9342	0.876	0.786	0.765	0.889	0.765	0.876	0.817	0.467	train
Nomogram	0.874	0.909	0.8377–0.9805	0.837	0.909	0.900	0.851	0.900	0.837	0.867	0.577	validation
HIP_MLP	0.816	0.834	0.7435–0.9235	0.698	0.932	0.909	0.759	0.909	0.698	0.789	0.436	validation
HIP1_MLP	0.759	0.776	0.6764–0.8765	0.744	0.773	0.762	0.756	0.762	0.744	0.753	0.338	validation
HIP2_MLP	0.805	0.832	0.7413–0.9225	0.744	0.864	0.842	0.776	0.842	0.744	0.790	0.445	validation
HIP3_MLP	0.770	0.817	0.7251–0.9091	0.767	0.773	0.767	0.773	0.767	0.767	0.767	0.223	validation
peri1_MLP	0.678	0.721	0.6134–0.8285	0.628	0.727	0.692	0.667	0.692	0.628	0.659	0.465	validation
peri2_SVM	0.678	0.710	0.6003–0.8199	0.674	0.682	0.674	0.682	0.674	0.674	0.674	0.457	validation
peri3_SVM	0.678	0.686	0.5711–0.7999	0.721	0.636	0.660	0.700	0.660	0.721	0.689	0.434	validation
int_MLP	0.770	0.797	0.6988–0.8942	0.791	0.750	0.756	0.786	0.756	0.791	0.773	0.399	validation
Ha1_LR	0.644	0.663	0.5479–0.7777	0.349	0.932	0.833	0.594	0.833	0.349	0.492	0.542	validation
Ha2_LR	0.678	0.717	0.6101–0.8243	0.465	0.886	0.800	0.629	0.800	0.465	0.588	0.578	validation
Ha3_SVM	0.736	0.795	0.7016–0.8893	0.860	0.614	0.685	0.818	0.685	0.860	0.763	0.386	validation
Nomogram	0.810	0.889	0.8212–0.9561	0.727	0.900	0.889	0.750	0.889	0.727	0.800	0.530	external test
HIP_MLP	0.845	0.869	0.7863–0.9524	0.818	0.875	0.878	0.814	0.878	0.818	0.847	0.339	external test
HIP1_MLP	0.798	0.838	0.7521–0.9241	0.886	0.700	0.765	0.848	0.765	0.886	0.821	0.207	external test
HIP2_MLP	0.821	0.853	0.7671–0.9385	0.773	0.875	0.872	0.778	0.872	0.773	0.819	0.480	external test
HIP3_MLP	0.798	0.870	0.7944–0.9454	0.864	0.725	0.776	0.829	0.776	0.864	0.817	0.182	external test
peri1_MLP	0.702	0.718	0.6071–0.8281	0.568	0.850	0.806	0.642	0.806	0.568	0.667	0.564	external test
peri2_SVM	0.726	0.735	0.6267–0.8438	0.750	0.700	0.733	0.718	0.733	0.750	0.742	0.438	external test
peri3_SVM	0.702	0.735	0.6266–0.8439	0.614	0.800	0.771	0.653	0.771	0.614	0.684	0.484	external test
int_MLP	0.762	0.781	0.6787–0.8838	0.795	0.725	0.761	0.763	0.761	0.795	0.778	0.379	external test
Ha1_LR	0.667	0.667	0.5489–0.7852	0.727	0.600	0.667	0.667	0.667	0.727	0.696	0.408	external test
Ha2_LR	0.738	0.743	0.6337–0.8515	0.886	0.575	0.696	0.821	0.696	0.886	0.780	0.345	external test
Ha3_SVM	0.774	0.823	0.7308–0.9146	0.705	0.850	0.838	0.723	0.838	0.705	0.765	0.476	external test

### Model explainability analysis

**Fig. 6:**
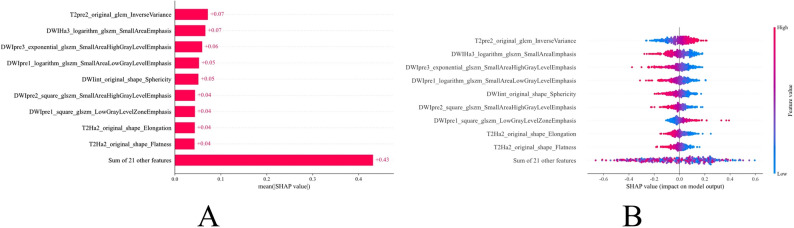
SHAP-based interpretability analysis of the predictive model

This study introduced the SHAP (SHapley Additive exPlanations) framework to perform an in-depth interpretability analysis of the optimal HIP3 predictive model. The SHAP summary plot (Fig. 6A) ranks features by their average SHAP values in descending order, revealing that T2pre2_original_glcm_InverseVariance has the highest contribution to predicting lymphovascular invasion (LVI) in colorectal cancer, followed by DWIHa3_logarithm_glszm_SmallAreaEmphasis and DWIpre3_exponential_glszm_SmallAreaHighGrayLevelEmphasis. The SHAP beeswarm plot (Fig. 6B) displays each sample’s SHAP value alongside its feature value, with colors ranging from blue (low value) to red (high value). The SHAP values on the horizontal axis represent the predicted probability of LVI positivity; higher SHAP values indicate a greater likelihood that the model predicts the patient as LVI positive. This visualization intuitively demonstrates the model’s decision-making process and the specific contribution of each feature to the prediction, enhancing the model’s interpretability and clinical reliability.

## Discussion

The HIP fusion model proposed in this study integrates clinical risk factors with multiscale radiomics information from Habitat, intra-tumoral, and peri-tumoral regions, ultimately constructing an intuitive and user-friendly nomogram. The model demonstrated excellent predictive performance and good calibration across multiple cohorts, proving its significant clinical potential for guiding personalized treatment decisions in rectal cancer patients.

MRI-based radiomics has become a hot research area for non-invasive prediction of lymphovascular invasion (LVI), with numerous studies confirming its feasibility and effectiveness across various tumors. Wang et al. [27] developed an MRI radiomics model for endometrial cancer, achieving AUC of 0.899 and 0.812 in the training and validation cohorts for LVI prediction, respectively. Their combined model integrating clinical features improved the AUC to 0.934 and 0.905 in the two cohorts. Zhang et al. [11] used a multimodal radiomics model to predict LVI in rectal cancer, with AUC of 0.884 in the training set and 0.876 in the validation set. Furthermore, models combining features from both intra-tumoral and peri-tumoral regions showed superior predictive performance. Zhao et al. [28] reported in invasive breast cancer that a support vector machine model integrating intra-tumoral and peri-tumoral features achieved AUC of 0.921 and 0.906 in training and validation sets, respectively. This study further highlights the significant value of radiomic features derived from different peritumoral regions in predicting lymphovascular invasion (LVI) in rectal cancer. Specifically, we evaluated the predictive performance of peritumoral subregions located 0–2 mm (Peri1), 2–4 mm (Peri2), and 4–6 mm (Peri3) from the tumor boundary. The results demonstrated that the Peri1 model achieved particularly favorable performance, with AUCs of 0.855, 0.721, and 0.718 in the training, internal validation, and external testing cohorts, respectively. These findings suggest that radiomic features extracted from the Peri1 region can consistently capture the complex interactions between the tumor and its surrounding microenvironment, thereby conferring strong predictive power for LVI. This finding not only confirms the critical role of the peri-tumoral region in tumor biological behavior but also highlights the importance of detailed analysis of different peri-tumoral layers for improving subregional predictive model performance.

In recent years, Habitat radiomics has demonstrated significant advantages in predictive analyses across multiple tumor types. By finely segmenting different subregions within the tumor, it can more precisely reflect the heterogeneity of the tumor microenvironment and its association with clinical outcomes. In a study by Zhu et al. [29] on nasopharyngeal carcinoma patients, a predictive model for neoadjuvant chemotherapy response based on Habitat subregion features from multimodal MRI achieved AUCs of 0.88 and 0.85 in the training and validation sets, respectively, significantly outperforming single-modality models. Additionally, for esophageal cancer patients, Kong et al. [30] employed a CT-based Habitat radiomics model to predict the efficacy of neoadjuvant chemotherapy and immunotherapy, with a multicenter validated AUC of 0.87, demonstrating good generalizability and clinical potential. In hepatocellular carcinoma, Sui et al. [31] developed a prognostic model using 18 F-FDG PET/CT combined with Habitat features and machine learning, with a 1-year progression-free survival AUC of 0.90, further underscoring the value of Habitat analysis in prognosis prediction. Although research on predicting lymphovascular invasion (LVI) is still limited, Habitat radiomics excels in revealing the complex spatial heterogeneity between tumor interior and peritumoral microenvironment. This study systematically constructed an MRI-based HIP model integrating multiple tumor microenvironment subregion features, significantly improving LVI prediction accuracy. The model performed excellently in preoperative LVI prediction for rectal cancer, showing higher AUC and greater stability than traditional intratumoral models, providing new technical support for personalized precision diagnosis and treatment.

Although machine learning models have achieved great success in medical image analysis, their “black-box” nature significantly limits their credibility and acceptance in clinical decision-making [32]. To address this issue, this study employed the SHAP (SHapley Additive exPlanations) analysis framework to enhance the model’s interpretability. SHAP quantifies the contribution of each feature to an individual prediction, thereby revealing the model’s decision logic [33]. The SHAP analysis not only identified the key features contributing most to LVI prediction at the global level (such as T2pre2_original_glcm_InverseVariance) but also revealed the positive or negative relationships between these features and LVI risk. More importantly, SHAP can generate personalized explanation plots for each patient, intuitively demonstrating the imaging clues upon which the model bases its classification of the patient as high-risk or low-risk. This individualized explanatory capability is crucial for clinicians, as it enables them not only to know the model’s prediction results but also to understand the underlying “imaging evidence,” thereby enhancing trust in the model’s predictions and facilitating better communication with patients about treatment plans, promoting precision medicine assisted by AI.

Despite the positive outcomes of this study, some limitations remain. First, this study is inherently retrospective. Although we employed rigorous internal and external validations to assess the model’s robustness, prospective, multicenter, large-sample clinical trials remain the gold standard to verify its clinical utility. Second, the tumor and peritumoral regions were delineated using a semi-automatic segmentation method, which inevitably introduces inter-observer variability. In the future, the development of fully automatic, high-precision deep learning segmentation algorithms will be key to improving the reproducibility and clinical applicability of the research. Third, this study only included the T2WI and DWI MRI sequences; future work could attempt to integrate more functional imaging sequences (such as dynamic contrast-enhanced DCE-MRI) or multimodal data (such as clinicopathological and genomic data) to construct multimodal and multi-omics predictive models, potentially further improving prediction accuracy. Finally, future prospective studies should evaluate the model’s performance in neoadjuvant-treated patients, potentially incorporating post-treatment imaging features and response assessment parameters. Understanding lymphovascular invasion prediction in treatment-naïve patients provides the essential foundation for developing more complex models applicable to post-neoadjuvant scenarios.

## Conclusion

In summary, this study successfully developed and validated a novel nomogram model based on multi-scale MRI radiomics for the noninvasive preoperative prediction of lymphovascular invasion (LVI) status in patients with rectal cancer. The model achieved excellent predictive performance and good clinical applicability by deeply integrating quantitative features from Habitat, intratumoral, and peritumoral regions along with key clinical risk factors. Moreover, the application of SHAP analysis revealed the internal decision-making mechanism of the model, enhancing its transparency and credibility. This radiomics tool, validated across multiple centers, holds promise to become a powerful aid for clinicians in preoperative risk stratification and individualized treatment planning, ultimately improving the prognosis for patients with rectal cancer.

## Supplementary Information

Below is the link to the electronic supplementary material.

Supplementary Material 1

## Data Availability

The datasets used and/or analysed during the current study are available from the corresponding author on reasonable request.

## Abbreviations

RC: Rectal cancer
FOBT: Fecal occult blood test
HBP: High blood pressure
CEA: Carcinoembryonic antigen
MRI: Magnetic resonance imaging
T2WI: T2-weighted imaging
DWI: Diffusion-weighted imaging
LVI: Lymphovascular invasion
SHAP: SHapley Additive exPlanations
ROC: Receiver operating characteristic
DCA: Decision curve analysis
AUC: Area under the curve
ICC: Intraclass correlation coefficients
ROI: Regions of interest
