# Supplementary material for: Integrative multi-region MRI radiomics and clinical nomogram for preoperative lymphovascular invasion prediction in rectal cancer: a multicenter validation
Source: BMC Med Imaging. 2025 Dec 19;26:48. doi: 10.1186/s12880-025-02105-1 (PMC12831238; doi:10.1186/s12880-025-02105-1)
Supplement: Supplementary file 1 — Supplementary Material 1 [file 12880_2025_2105_MOESM1_ESM.docx]

Supplementary Material

# S1. The detailed imaging parameters

The scanning parameters for the Siemens Skyra 3.0T MRI are set as follows: (1) Axial T2-weighted imaging (T2WI) sequence: Field of View (FOV) 220 mm × 220 mm, matrix 256 × 256, Echo Time (TE) 110 ms, Repetition Time (TR) 5000 ms, slice thickness 5 mm; (2) Axial Diffusion-Weighted Imaging (DWI) sequence: b-value = 800-1000 s/mm², Field of View (FOV) 340 mm × 300 mm, matrix 256 × 256, Echo Time (TE) 60 ms, Repetition Time (TR) 3000 ms, slice thickness 5 mm.

The scanning parameters for the 3.0T General Electric (GE) MRI are set as follows: (1) Axial T2-weighted imaging (T2WI) sequence: Field of View (FOV) 240 mm × 240 mm, matrix 256 × 256, Echo Time (TE) 120 ms, Repetition Time (TR) 5000 ms, slice thickness 5 mm; (2) Axial Diffusion-Weighted Imaging (DWI) sequence: b-value = 800-1000 s/mm², Field of View (FOV) 320 mm × 320 mm, matrix 256 × 256, Echo Time (TE) 60 ms, Repetition Time (TR) 3000 ms, slice thickness 5 mm.

# Habitat clustering features

In this study, we extracted 19 radiomic features from each voxel within the tumor ROI to comprehensively capture local signal intensity and texture variations. These features included: firstorder_Median, firstorder_Entropy, firstorder_MeanAbsoluteDeviation, glcm_JointEntropy, glcm_DifferenceVariance, glcm_Imc1, glcm_SumEntropy, glcm_InverseVariance, glcm_DifferenceAverage, glcm_Imc2, glcm_JointEnergy, glcm_DifferenceEntropy, glszm_SmallAreaHighGrayLevelEmphasis, glszm_SizeZoneNonUniformityNormalized, glrlm_RunVariance, glrlm_RunEntropy, glrlm_LongRunEmphasis, ngtdm_Strength and ngtdm_Contrast.

# Supplementary Figures and Tables

## Supplementary Figures


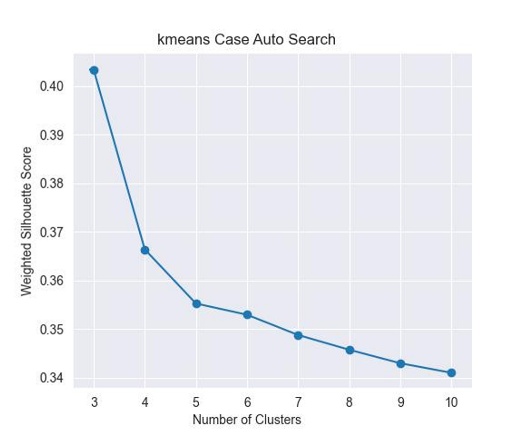


**Supplementary Figure 1.** An unsupervised K-means clustering method was applied to the pixel values, with the number of clusters ranging from 3 to 10. For each potential cluster number, the K-means algorithm was executed, and the corresponding Silhouette Score was recorded. A higher Silhouette Score indicates better separation between clusters relative to cohesion within clusters. Pixels assigned to the same cluster were given the same color to generate clustering labels. Based on the Silhouette Score values, the optimal number of clusters was determined to be three.

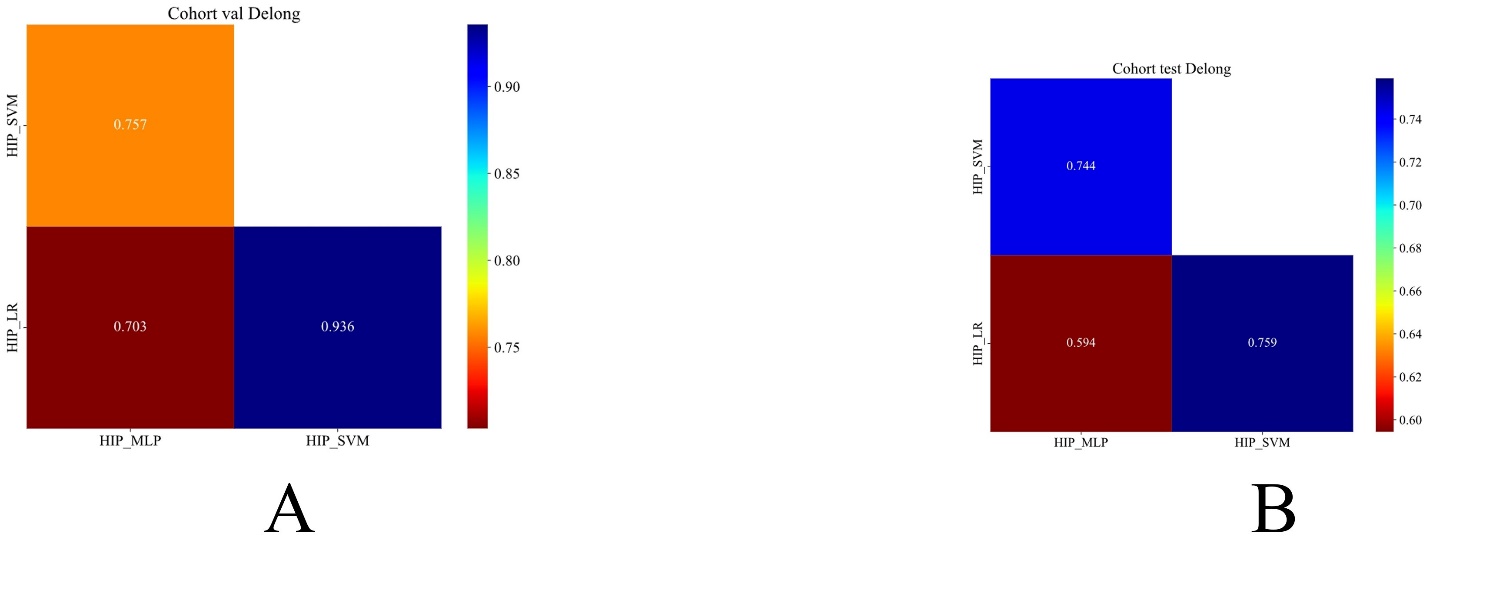


**Supplementary Figure 2.** DeLong test results: comparison of the classification performance of different HIP algorithms for LVI in the internal validation set (A) and the external test set (B)

## Supplementary Tables

S2. Hyperparameters of each model.

| Signature | probability | max_iter | kernel | Signature | hidden_layer_sizes | max_iter | solver | random_state | Signature | penalty | max_iter |
| --- | --- | --- | --- | --- | --- | --- | --- | --- | --- | --- | --- |
| HIP_SVM | TRUE | 65 | rbf | HIP_MLP | (128,) | 1465 | adam | 0 | HIP_LR | l2 | 315 |
| HIP1_SVM | TRUE | 437 | linear | HIP1_MLP | (512,) | 170 | adam | 0 | HIP1_LR | l2 | 6 |
| HIP2_SVM | TRUE | 383 | linear | HIP2_MLP | (16,) | 1471 | adam | 0 | HIP2_LR | l2 | 6 |
| HIP3_SVM | TRUE | 74 | rbf | HIP3_MLP | (128, 64,32) | 85 | adam | 0 | HIP3_LR | l2 | 315 |
| peri1_SVM | TRUE | 411 | linear | peri1_MLP | (64, 32) | 757 | sgd | 0 | peri1_LR | l2 | 3 |
| peri2_SVM | TRUE | 417 | linear | peri2_MLP | (512, 256) | 1083 | sgd | 0 | peri2_LR | l2 | 6 |
| peri3_SVM | TRUE | 247 | linear | peri3_MLP | (128, 64) | 1313 | sgd | 0 | peri3_LR | l2 | 315 |
| int_SVM | TRUE | 60 | rbf | int_MLP | (256, 128, 64) | 820 | sgd | 0 | int_LR | l2 | 3 |
| Ha1_SVM | TRUE | 101 | rbf | Ha1_MLP | (256, 128, 64) | 697 | adam | 0 | Ha1_LR | l2 | 6 |
| Ha2_SVM | TRUE | 68 | rbf | Ha2_MLP | (256, 128, 64) | 820 | sgd | 0 | Ha2_LR | l2 | 6 |
| Ha3_SVM | TRUE | 79 | rbf | Ha3_MLP | (256,) | 1168 | adam | 0 | Ha3_LR | l2 | 17 |

S3. Comparative performance of each radiomics model across three machine learning algorithms: multilayer perceptron (MLP), support vector machine (SVM), and logistic regression (LR)

| Signature | Accuracy | AUC | 95% CI | Sensitivity | Specificity | PPV | NPV | Precision | Recall | F1 | Threshold | Cohort |
| --- | --- | --- | --- | --- | --- | --- | --- | --- | --- | --- | --- | --- |
| HIP_MLP | 0.886 | 0.953 | 0.9276 - 0.9785 | 0.933 | 0.848 | 0.830 | 0.941 | 0.830 | 0.933 | 0.878 | 0.291 | train |
| HIP1_MLP | 0.786 | 0.873 | 0.8269 - 0.9191 | 0.742 | 0.821 | 0.767 | 0.800 | 0.767 | 0.742 | 0.754 | 0.353 | train |
| HIP2_MLP | 0.881 | 0.932 | 0.8983 - 0.9648 | 0.921 | 0.848 | 0.828 | 0.931 | 0.828 | 0.921 | 0.872 | 0.417 | train |
| HIP3_MLP | 0.841 | 0.921 | 0.8862 - 0.9557 | 0.910 | 0.786 | 0.771 | 0.917 | 0.771 | 0.910 | 0.835 | 0.168 | train |
| peri1_MLP | 0.786 | 0.855 | 0.8050 - 0.9059 | 0.708 | 0.848 | 0.787 | 0.785 | 0.787 | 0.708 | 0.746 | 0.491 | train |
| peri2_MLP | 0.766 | 0.819 | 0.7610 - 0.8762 | 0.640 | 0.866 | 0.792 | 0.752 | 0.792 | 0.640 | 0.708 | 0.613 | train |
| peri3_MLP | 0.701 | 0.733 | 0.6636 - 0.8019 | 0.663 | 0.732 | 0.663 | 0.732 | 0.663 | 0.663 | 0.663 | 0.498 | train |
| int_MLP | 0.776 | 0.813 | 0.7545 - 0.8723 | 0.787 | 0.768 | 0.729 | 0.819 | 0.729 | 0.787 | 0.757 | 0.396 | train |
| Ha1_MLP | 0.672 | 0.712 | 0.6417 - 0.7833 | 0.640 | 0.696 | 0.626 | 0.709 | 0.626 | 0.640 | 0.633 | 0.362 | train |
| Ha2_MLP | 0.682 | 0.740 | 0.6716 - 0.8085 | 0.708 | 0.661 | 0.624 | 0.740 | 0.624 | 0.708 | 0.663 | 0.400 | train |
| Ha3_MLP | 0.811 | 0.881 | 0.8343 - 0.9283 | 0.854 | 0.777 | 0.752 | 0.870 | 0.752 | 0.854 | 0.800 | 0.418 | train |
| HIP_MLP | 0.816 | 0.834 | 0.7435 - 0.9235 | 0.698 | 0.932 | 0.909 | 0.759 | 0.909 | 0.698 | 0.789 | 0.436 | validation |
| HIP1_MLP | 0.759 | 0.776 | 0.6764 - 0.8765 | 0.744 | 0.773 | 0.762 | 0.756 | 0.762 | 0.744 | 0.753 | 0.338 | validation |
| HIP2_MLP | 0.805 | 0.832 | 0.7413 - 0.9225 | 0.744 | 0.864 | 0.842 | 0.776 | 0.842 | 0.744 | 0.790 | 0.445 | validation |
| HIP3_MLP | 0.770 | 0.817 | 0.7251 - 0.9091 | 0.767 | 0.773 | 0.767 | 0.773 | 0.767 | 0.767 | 0.767 | 0.223 | validation |
| peri1_MLP | 0.678 | 0.721 | 0.6134 - 0.8285 | 0.628 | 0.727 | 0.692 | 0.667 | 0.692 | 0.628 | 0.659 | 0.465 | validation |
| peri2_MLP | 0.667 | 0.691 | 0.5785 - 0.8031 | 0.698 | 0.636 | 0.652 | 0.683 | 0.652 | 0.698 | 0.674 | 0.551 | validation |
| peri3_MLP | 0.655 | 0.673 | 0.5575 - 0.7882 | 0.419 | 0.886 | 0.783 | 0.609 | 0.783 | 0.419 | 0.545 | 0.576 | validation |
| int_MLP | 0.770 | 0.797 | 0.6988 - 0.8942 | 0.791 | 0.750 | 0.756 | 0.786 | 0.756 | 0.791 | 0.773 | 0.399 | validation |
| Ha1_MLP | 0.632 | 0.635 | 0.5181 - 0.7525 | 0.395 | 0.864 | 0.739 | 0.594 | 0.739 | 0.395 | 0.515 | 0.441 | validation |
| Ha2_MLP | 0.678 | 0.693 | 0.5819 - 0.8039 | 0.581 | 0.773 | 0.714 | 0.654 | 0.714 | 0.581 | 0.641 | 0.426 | validation |
| Ha3_MLP | 0.736 | 0.753 | 0.6489 - 0.8564 | 0.674 | 0.795 | 0.763 | 0.714 | 0.763 | 0.674 | 0.716 | 0.477 | validation |
| HIP_MLP | 0.845 | 0.869 | 0.7863 - 0.9524 | 0.818 | 0.875 | 0.878 | 0.814 | 0.878 | 0.818 | 0.847 | 0.339 | external test |
| HIP1_MLP | 0.798 | 0.838 | 0.7521 - 0.9241 | 0.886 | 0.700 | 0.765 | 0.848 | 0.765 | 0.886 | 0.821 | 0.207 | external test |
| HIP2_MLP | 0.821 | 0.853 | 0.7671 - 0.9385 | 0.773 | 0.875 | 0.872 | 0.778 | 0.872 | 0.773 | 0.819 | 0.480 | external test |
| HIP3_MLP | 0.798 | 0.870 | 0.7944 - 0.9454 | 0.864 | 0.725 | 0.776 | 0.829 | 0.776 | 0.864 | 0.817 | 0.182 | external test |
| peri1_MLP | 0.702 | 0.718 | 0.6071 - 0.8281 | 0.568 | 0.850 | 0.806 | 0.642 | 0.806 | 0.568 | 0.667 | 0.564 | external test |
| peri2_MLP | 0.702 | 0.723 | 0.6126 - 0.8340 | 0.795 | 0.600 | 0.686 | 0.727 | 0.686 | 0.795 | 0.737 | 0.500 | external test |
| peri3_MLP | 0.679 | 0.731 | 0.6236 - 0.8377 | 0.500 | 0.875 | 0.815 | 0.614 | 0.815 | 0.500 | 0.620 | 0.573 | external test |
| int_MLP | 0.762 | 0.781 | 0.6787 - 0.8838 | 0.795 | 0.725 | 0.761 | 0.763 | 0.761 | 0.795 | 0.778 | 0.379 | external test |
| Ha1_MLP | 0.655 | 0.661 | 0.5438 - 0.7778 | 0.659 | 0.650 | 0.674 | 0.634 | 0.674 | 0.659 | 0.667 | 0.352 | external test |
| Ha2_MLP | 0.667 | 0.714 | 0.6037 - 0.8247 | 0.500 | 0.850 | 0.786 | 0.607 | 0.786 | 0.500 | 0.611 | 0.439 | external test |
| Ha3_MLP | 0.821 | 0.878 | 0.8032 - 0.9536 | 0.864 | 0.775 | 0.809 | 0.838 | 0.809 | 0.864 | 0.835 | 0.312 | external test |

| Signature | Accuracy | AUC | 95% CI | Sensitivity | Specificity | PPV | NPV | Precision | Recall | F1 | Threshold | Cohort |
| --- | --- | --- | --- | --- | --- | --- | --- | --- | --- | --- | --- | --- |
| HIP_SVM | 0.910 | 0.963 | 0.9369 - 0.9887 | 0.876 | 0.937 | 0.918 | 0.905 | 0.918 | 0.876 | 0.897 | 0.535 | train |
| HIP1_SVM | 0.786 | 0.840 | 0.7859 - 0.8941 | 0.663 | 0.884 | 0.819 | 0.767 | 0.819 | 0.663 | 0.733 | 0.548 | train |
| HIP2_SVM | 0.801 | 0.879 | 0.8317 - 0.9256 | 0.820 | 0.786 | 0.753 | 0.846 | 0.753 | 0.820 | 0.785 | 0.467 | train |
| HIP3_SVM | 0.886 | 0.944 | 0.9128 - 0.9761 | 0.933 | 0.848 | 0.830 | 0.941 | 0.830 | 0.933 | 0.878 | 0.402 | train |
| peri1_SVM | 0.721 | 0.775 | 0.7122 - 0.8387 | 0.584 | 0.830 | 0.732 | 0.715 | 0.732 | 0.584 | 0.650 | 0.495 | train |
| peri2_SVM | 0.731 | 0.792 | 0.7297 - 0.8539 | 0.753 | 0.714 | 0.677 | 0.784 | 0.677 | 0.753 | 0.713 | 0.453 | train |
| peri3_SVM | 0.652 | 0.711 | 0.6402 - 0.7818 | 0.820 | 0.518 | 0.575 | 0.784 | 0.575 | 0.820 | 0.676 | 0.396 | train |
| int_SVM | 0.761 | 0.846 | 0.7932 - 0.8986 | 0.798 | 0.732 | 0.703 | 0.820 | 0.703 | 0.798 | 0.747 | 0.459 | train |
| Ha1_SVM | 0.687 | 0.758 | 0.6917 - 0.8241 | 0.787 | 0.607 | 0.614 | 0.782 | 0.614 | 0.787 | 0.690 | 0.417 | train |
| Ha2_SVM | 0.736 | 0.792 | 0.7287 - 0.8546 | 0.663 | 0.795 | 0.720 | 0.748 | 0.720 | 0.663 | 0.690 | 0.462 | train |
| Ha3_SVM | 0.826 | 0.889 | 0.8441 - 0.9342 | 0.876 | 0.786 | 0.765 | 0.889 | 0.765 | 0.876 | 0.817 | 0.467 | train |
| HIP_SVM | 0.782 | 0.825 | 0.7356 - 0.9150 | 0.674 | 0.886 | 0.853 | 0.736 | 0.853 | 0.674 | 0.753 | 0.516 | validation |
| HIP1_SVM | 0.724 | 0.754 | 0.6503 - 0.8571 | 0.651 | 0.795 | 0.757 | 0.700 | 0.757 | 0.651 | 0.700 | 0.482 | validation |
| HIP2_SVM | 0.770 | 0.820 | 0.7308 - 0.9098 | 0.884 | 0.659 | 0.717 | 0.853 | 0.717 | 0.884 | 0.792 | 0.410 | validation |
| HIP3_SVM | 0.782 | 0.793 | 0.6943 - 0.8924 | 0.628 | 0.932 | 0.900 | 0.719 | 0.900 | 0.628 | 0.740 | 0.580 | validation |
| peri1_SVM | 0.690 | 0.715 | 0.6066 - 0.8242 | 0.628 | 0.750 | 0.711 | 0.673 | 0.711 | 0.628 | 0.667 | 0.480 | validation |
| peri2_SVM | 0.678 | 0.710 | 0.6003 - 0.8199 | 0.674 | 0.682 | 0.674 | 0.682 | 0.674 | 0.674 | 0.674 | 0.457 | validation |
| peri3_SVM | 0.678 | 0.686 | 0.5711 - 0.7999 | 0.721 | 0.636 | 0.660 | 0.700 | 0.660 | 0.721 | 0.689 | 0.434 | validation |
| int_SVM | 0.713 | 0.744 | 0.6393 - 0.8491 | 0.605 | 0.818 | 0.765 | 0.679 | 0.765 | 0.605 | 0.675 | 0.524 | validation |
| Ha1_SVM | 0.598 | 0.632 | 0.5146 - 0.7486 | 0.535 | 0.659 | 0.605 | 0.592 | 0.605 | 0.535 | 0.568 | 0.447 | validation |
| Ha2_SVM | 0.678 | 0.656 | 0.5378 - 0.7740 | 0.628 | 0.727 | 0.692 | 0.667 | 0.692 | 0.628 | 0.659 | 0.458 | validation |
| Ha3_SVM | 0.736 | 0.795 | 0.7016 - 0.8893 | 0.860 | 0.614 | 0.685 | 0.818 | 0.685 | 0.860 | 0.763 | 0.386 | validation |
| HIP_SVM | 0.857 | 0.874 | 0.7923 - 0.9565 | 0.818 | 0.900 | 0.900 | 0.818 | 0.900 | 0.818 | 0.857 | 0.511 | external test |
| HIP1_SVM | 0.833 | 0.830 | 0.7345 - 0.9246 | 0.932 | 0.725 | 0.788 | 0.906 | 0.788 | 0.932 | 0.854 | 0.389 | external test |
| HIP2_SVM | 0.798 | 0.813 | 0.7163 - 0.9099 | 0.841 | 0.750 | 0.787 | 0.811 | 0.787 | 0.841 | 0.813 | 0.464 | external test |
| HIP3_SVM | 0.810 | 0.846 | 0.7619 - 0.9302 | 0.886 | 0.725 | 0.780 | 0.853 | 0.780 | 0.886 | 0.830 | 0.419 | external test |
| peri1_SVM | 0.690 | 0.705 | 0.5931 - 0.8160 | 0.955 | 0.400 | 0.636 | 0.889 | 0.636 | 0.955 | 0.764 | 0.334 | external test |
| peri2_SVM | 0.726 | 0.735 | 0.6267 - 0.8438 | 0.750 | 0.700 | 0.733 | 0.718 | 0.733 | 0.750 | 0.742 | 0.438 | external test |
| peri3_SVM | 0.702 | 0.735 | 0.6266 - 0.8439 | 0.614 | 0.800 | 0.771 | 0.653 | 0.771 | 0.614 | 0.684 | 0.484 | external test |
| int_SVM | 0.762 | 0.802 | 0.7056 - 0.8978 | 0.909 | 0.600 | 0.714 | 0.857 | 0.714 | 0.909 | 0.800 | 0.422 | external test |
| Ha1_SVM | 0.667 | 0.678 | 0.5601 - 0.7950 | 0.773 | 0.550 | 0.654 | 0.687 | 0.654 | 0.773 | 0.708 | 0.414 | external test |
| Ha2_SVM | 0.702 | 0.691 | 0.5770 - 0.8060 | 0.636 | 0.775 | 0.757 | 0.660 | 0.757 | 0.636 | 0.691 | 0.456 | external test |
| Ha3_SVM | 0.774 | 0.823 | 0.7308 - 0.9146 | 0.705 | 0.850 | 0.838 | 0.723 | 0.838 | 0.705 | 0.765 | 0.476 | external test |

| Signature | Accuracy | AUC | 95% CI | Sensitivity | Specificity | PPV | NPV | Precision | Recall | F1 | Threshold | Cohort |
| --- | --- | --- | --- | --- | --- | --- | --- | --- | --- | --- | --- | --- |
| HIP_LR | 0.841 | 0.917 | 0.8795 - 0.9546 | 0.831 | 0.848 | 0.813 | 0.864 | 0.813 | 0.831 | 0.822 | 0.411 | train |
| HIP1_LR | 0.776 | 0.844 | 0.7917 - 0.8965 | 0.618 | 0.902 | 0.833 | 0.748 | 0.833 | 0.618 | 0.710 | 0.599 | train |
| HIP2_LR | 0.841 | 0.901 | 0.8594 - 0.9418 | 0.843 | 0.839 | 0.806 | 0.870 | 0.806 | 0.843 | 0.824 | 0.409 | train |
| HIP3_LR | 0.781 | 0.857 | 0.8070 - 0.9077 | 0.798 | 0.768 | 0.732 | 0.827 | 0.732 | 0.798 | 0.763 | 0.438 | train |
| peri1_LR | 0.731 | 0.790 | 0.7283 - 0.8524 | 0.775 | 0.696 | 0.670 | 0.796 | 0.670 | 0.775 | 0.719 | 0.409 | train |
| peri2_LR | 0.736 | 0.796 | 0.7348 - 0.8569 | 0.719 | 0.750 | 0.696 | 0.771 | 0.696 | 0.719 | 0.707 | 0.448 | train |
| peri3_LR | 0.662 | 0.713 | 0.6420 - 0.7834 | 0.775 | 0.571 | 0.590 | 0.762 | 0.590 | 0.775 | 0.670 | 0.398 | train |
| int_LR | 0.731 | 0.766 | 0.6998 - 0.8317 | 0.708 | 0.750 | 0.692 | 0.764 | 0.692 | 0.708 | 0.700 | 0.465 | train |
| Ha1_LR | 0.647 | 0.663 | 0.5874 - 0.7384 | 0.584 | 0.696 | 0.605 | 0.678 | 0.605 | 0.584 | 0.594 | 0.465 | train |
| Ha2_LR | 0.692 | 0.720 | 0.6497 - 0.7911 | 0.764 | 0.634 | 0.624 | 0.772 | 0.624 | 0.764 | 0.687 | 0.425 | train |
| Ha3_LR | 0.796 | 0.841 | 0.7861 - 0.8963 | 0.753 | 0.830 | 0.779 | 0.809 | 0.779 | 0.753 | 0.766 | 0.473 | train |
| HIP_LR | 0.828 | 0.828 | 0.7319 - 0.9235 | 0.744 | 0.909 | 0.889 | 0.784 | 0.889 | 0.744 | 0.810 | 0.516 | validation |
| HIP1_LR | 0.713 | 0.742 | 0.6367 - 0.8464 | 0.651 | 0.773 | 0.737 | 0.694 | 0.737 | 0.651 | 0.691 | 0.466 | validation |
| HIP2_LR | 0.759 | 0.780 | 0.6824 - 0.8778 | 0.558 | 0.955 | 0.923 | 0.689 | 0.923 | 0.558 | 0.696 | 0.647 | validation |
| HIP3_LR | 0.782 | 0.788 | 0.6889 - 0.8872 | 0.674 | 0.886 | 0.853 | 0.736 | 0.853 | 0.674 | 0.753 | 0.566 | validation |
| peri1_LR | 0.678 | 0.710 | 0.6007 - 0.8190 | 0.767 | 0.591 | 0.647 | 0.722 | 0.647 | 0.767 | 0.702 | 0.344 | validation |
| peri2_LR | 0.644 | 0.678 | 0.5648 - 0.7915 | 0.767 | 0.523 | 0.611 | 0.697 | 0.611 | 0.767 | 0.680 | 0.311 | validation |
| peri3_LR | 0.667 | 0.674 | 0.5598 - 0.7891 | 0.651 | 0.682 | 0.667 | 0.667 | 0.667 | 0.651 | 0.659 | 0.465 | validation |
| int_LR | 0.736 | 0.749 | 0.6441 - 0.8538 | 0.721 | 0.750 | 0.738 | 0.733 | 0.738 | 0.721 | 0.729 | 0.434 | validation |
| Ha1_LR | 0.644 | 0.663 | 0.5479 - 0.7777 | 0.349 | 0.932 | 0.833 | 0.594 | 0.833 | 0.349 | 0.492 | 0.542 | validation |
| Ha2_LR | 0.678 | 0.717 | 0.6101 - 0.8243 | 0.465 | 0.886 | 0.800 | 0.629 | 0.800 | 0.465 | 0.588 | 0.578 | validation |
| Ha3_LR | 0.736 | 0.727 | 0.6160 - 0.8375 | 0.698 | 0.773 | 0.750 | 0.723 | 0.750 | 0.698 | 0.723 | 0.465 | validation |
| HIP_LR | 0.893 | 0.882 | 0.7985 - 0.9652 | 0.909 | 0.875 | 0.889 | 0.897 | 0.889 | 0.909 | 0.899 | 0.316 | external test |
| HIP1_LR | 0.810 | 0.827 | 0.7341 - 0.9205 | 0.841 | 0.775 | 0.804 | 0.816 | 0.804 | 0.841 | 0.822 | 0.396 | external test |
| HIP2_LR | 0.821 | 0.855 | 0.7694 - 0.9409 | 0.773 | 0.875 | 0.872 | 0.778 | 0.872 | 0.773 | 0.819 | 0.511 | external test |
| HIP3_LR | 0.833 | 0.886 | 0.8172 - 0.9556 | 0.818 | 0.850 | 0.857 | 0.810 | 0.857 | 0.818 | 0.837 | 0.466 | external test |
| peri1_LR | 0.667 | 0.690 | 0.5764 - 0.8042 | 0.886 | 0.425 | 0.629 | 0.773 | 0.629 | 0.886 | 0.736 | 0.251 | external test |
| peri2_LR | 0.690 | 0.709 | 0.5949 - 0.8222 | 0.636 | 0.750 | 0.737 | 0.652 | 0.737 | 0.636 | 0.683 | 0.455 | external test |
| peri3_LR | 0.702 | 0.747 | 0.6414 - 0.8518 | 0.773 | 0.625 | 0.694 | 0.714 | 0.694 | 0.773 | 0.731 | 0.379 | external test |
| int_LR | 0.798 | 0.801 | 0.7018 - 0.9005 | 0.864 | 0.725 | 0.776 | 0.829 | 0.776 | 0.864 | 0.817 | 0.400 | external test |
| Ha1_LR | 0.667 | 0.667 | 0.5489 - 0.7852 | 0.727 | 0.600 | 0.667 | 0.667 | 0.667 | 0.727 | 0.696 | 0.408 | external test |
| Ha2_LR | 0.738 | 0.743 | 0.6337 - 0.8515 | 0.886 | 0.575 | 0.696 | 0.821 | 0.696 | 0.886 | 0.780 | 0.345 | external test |
| Ha3_LR | 0.833 | 0.889 | 0.8168 - 0.9605 | 0.818 | 0.850 | 0.857 | 0.810 | 0.857 | 0.818 | 0.837 | 0.368 | external test |

S4 Composition factors of nomogram and relevant coefficients.
Nomogram = -4.7836+2.6808*N+8.1901*HIP，C-Indexes=0.978.
